# Impact of conservation tillage in rice–based cropping systems on soil aggregation, carbon pools and nutrients

**DOI:** 10.1016/j.geoderma.2019.01.001

**Published:** 2019-04-15

**Authors:** Rajiv Nandan, Vikram Singh, Sati Shankar Singh, Virender Kumar, Kali Krishna Hazra, Chaitanya Prasad Nath, Shishpal Poonia, Ram Kanwar Malik, Ranjan Bhattacharyya, Andrew McDonald

**Affiliations:** a Sam Higginbottom University of Agriculture Technology and Sciences (SHUATS), Allahabad, Uttar Pradesh, India; b ICAR–Indian Institute of Pulses Research (ICAR–IIPR), Kanpur, Uttar Pradesh, India; c International Rice Research Institute (IRRI), Los Baños, Laguna, Philippines; d International Maize and Wheat Improvement Center (CIMMYT), South Asia Regional Office, Patna, India; e ICAR–Indian Agricultural Research Institute, New Delhi, India; f International Maize and Wheat Improvement Center (CIMMYT), South Asia Regional Office, Kathmandu, Nepal

**Keywords:** Carbon fractions, Carbon stabilization, Grain yield, Soil aggregate, Soil available nutrients, Zero–till direct seeded rice

## Abstract

Tillage intensive cropping practices have deteriorated soil physical quality and decreased soil organic carbon (SOC) levels in rice–growing areas of South Asia. Consequently, crop productivity has declined over the years demonstrating the need for sustainable alternatives. Given that, a field experiment was conducted for six years to assess the impact of four tillage based crop establishment treatments [puddled transplant rice followed by conventional tillage in wheat/maize (CTTPR–CT), non–puddled transplant rice followed by zero–tillage in wheat/maize (NPTPR–ZT), zero–till transplant rice followed by zero–tillage in wheat/maize (ZTTPR–ZT), zero–tillage direct seeded rice followed by zero–tillage in wheat/maize (ZTDSR–ZT)], two residue management treatments [residue removal, residue retention (~33%)], and two cropping systems [rice–wheat, rice–maize] on soil aggregation, carbon pools, nutrient availability, and crop productivity. After six years of rotation, in top 0.2 m soil depth, zero–till crop establishment treatments (ZTTPR–ZT and ZTDSR–ZT) had higher (*p* < 0.05) total organic carbon (TOC) over conventional tillage treatment (CTTPR–CT). Zero–till crop establishment treatments increased very–labile C faction (C*frac*_1_) by 21% followed by labile fraction (C*frac*_2_) (16%), non–labile fraction (C*frac*_4_) (13%) and less–labile fraction (C*frac*_3_) (7%). Notably, higher passive C–pool in conservation tillage practices over CTTPR–CT suggests that conservation tillage could stabilize the recalcitrant form of carbon that persists longer in the soil. Meantime, zero–till crop establishment treatments had higher (*p* < 0.05) water stable macro–aggregates, macro–aggregates: micro–aggregates ratio and aggregate carbon content over CTTPR–CT. The treatment NPTPR–ZT significantly increased soil quality parameters over CTTPR–CT. However, the effect was not as prominent as that of ZTTPR–ZT and ZTDSR–ZT. Retention of crop residue increased (*p* < 0.05) TOC (12%) and soil available nutrients mainly available–P (16%), followed by available–K (12%), DTPA–extractable Zn (11%), and available–S (6%) over residue removal treatment. The constructive changes in soil properties following conservation tillage and crop residue retention led to increased crop productivity over conventional CTTPR–CT. Therefore, conservation tillage (particularly ZTTPR–ZT and ZTDSR–ZT) and crop residue retention could be recommended in tropical rice–based cropping systems for improving soil quality and production sustainability.

## Highlights

• Conservation tillage increased soil aggregation over conventional tillage.
• Zero-tillage treatments increased labile carbon stabilization over conventional tillage.
• Residue retention increased soil aggregation, soil available P, K and Zn over residue removal.
• Zero-tillage with residue retention increased crop yields over conventional tillage practice.

## 1 Introduction

The adverse impact of intensive tillage practices on soil physical quality and soil organic carbon (SOC) levels is a major challenge in tropical rice–growing regions (Chauhan et al., 2012; Srinivasan et al., 2012). Added to this, limited or no use of organic manures/crop residue (Ghosh et al., 2016), lack of crop diversification (Hazra et al., 2014), imbalanced use of mineral fertilizers (Brar et al., 2013) have further aggravated soil quality deterioration. In rice–growing regions, the long–term practice of puddling (wet tillage) affects soil aggregation, activity of beneficial microorganisms, and overall soil environments (Javurek and Vach, 2009; Pandey et al., 2012; Bhattacharyya et al., 2012). Conventional puddled transplanted rice management systems require more water and create ecologies that favour emission of methane – a potent greenhouse gas (Hou et al., 2000; Hazra and Chandra, 2016). In post–rainy season, conventional wheat/maize cultivation is also tillage–intensive, consisting of multiple passes of discs or tine harrows and planking for creating friable seedbed that leads to long turn–around periods between rice harvest and wheat/maize planting. So, alternative soil and crop management practices are needed to alleviate the adverse consequences of conventional puddled rice–based production systems and to remain sustainable in long–run.

In South Asia, the benefits of conservation tillage practices in resource conservation, soil quality and farm profitability have already been reported (Ladha et al., 2009; Gathala et al., 2013). However, the systematic research on conservation tillage in rice–based cropping systems is limited, particularly in the cropping system mode. Rice crop establishment with conservation tillage such as zero–tillage transplanting (ZTTPR), non–puddled transplanting (NPTPR), and ZT direct seeding of rice (ZTDSR) has developed as alternatives to conventional puddled transplanting rice (CTTPR) (Laik et al., 2014; Jat et al., 2014). Impact of these conservation tillage–based crop establishment practices with or without crop residue retention on soil aggregates, C in different aggregate size class, C–stabilization, and soil residual fertility has not adequately been addressed.

The lability–graded fractions of total organic carbon (TOC) provide valuable information related to the quality and persistence of soil organic carbon (Ghosh et al., 2012; Venkatesh et al., 2013). Primarily, tillage induces disruption of macro–aggregates and thus accelerates the SOC loss (Zotarelli et al., 2007; Andruschkewitsch et al., 2014a). Protection of organic carbon within stable soil aggregates is crucial for carbon stabilization and its persistence in soils. Thus, studying C–sequestration process is important for strategic SOC management, particularly in tropical rice soils, where native C stock is usually low.

Therefore, a field experiment (2009–2015) was conducted for six years to assess the effect of different tillage–based crop establishment treatments with or without crop residue retention under two rice–based production systems (rice–wheat and rice–winter maize) on soil aggregation, C–stabilization, and soil residual fertility. The specific objectives of the study were: (i) to assess the impact of conservation tillage based crop establishment practices and crop residue retention in rice–based production system on soil C dynamics, aggregate size fraction and aggregate–associated C content; (ii) to know the C–stabilization rate in different tillage based crop establishment practices in tropical rice–based cropping systems, and (iii) to assess the effect of crop rotation, residue retention, and tillage based crop establishment practices on soil residual fertility.

## 2 Materials and methods

### 2.1 Site and soil characteristics

The field experiment was conducted during 2009–2015 at the research farm of the Indian Council of Agricultural Research– Research Complex for Eastern Region (ICAR–RCER), Patna, Bihar (25°37′ N, 85°13′ E and 36 m above mean sea level). Climate of the site is subtropical humid. The mean annual rainfall of the area is 1130 mm. The experimental soil is silty–clay in texture and classified as Fluvisol (WRB soil classification. At the initiation of the experiment, the values of different soil parameters at surface soil depth (0–0.2 m) are given in Table 1.

**Table 1:** Soil physico–chemical properties (0–0.2 m) at the initiation of the experiment (2009).

Parameter	Value
Sand (%)	15.0
Silt (%)	41.0
Clay (%)	44.0
pH (1:2 soil: water)	7.11
EC (dS m^−1^)	0.38
Organic carbon (%)	0.49
Bulk density (g cm^−3^)	1.44
Penetration resistance (MPa)	1.75
Available–N (kg ha^−1^)	135.2
Available–P (kg ha^−1^)	35.2
Available–K (kg ha^−1^)	239.2
DTPA–extractable Zn (mg kg^−1^)	0.83
DTPA–extractable Fe (mg kg^−1^)	19.9
DTPA–extractable Mn (mg kg^−1^)	25.5
DTPA–extractable Cu (mg kg^−1^)	2.59

### 2.2 Treatment detail and experimental design

Treatments comprised of two levels of crop rotations [rice (*Oryza sativa* L.)–wheat (*Triticum aestivum* L.), rice–winter maize (*Zea mays* L.)], two levels of residue management treatments [residue removal, residue retention (~33%)], and four levels of tillage based crop establishment practices [conventional puddled transplanting of rice followed by conventional tillage (CT) in wheat/maize (CTTPR–CT); non–puddled transplanting of rice followed by zero tillage (ZT) in wheat/maize (NPTPR–ZT); ZT transplanting of rice followed by ZT in wheat/maize (ZTTPR–ZT); and ZT direct seeding of rice followed by ZT in wheat/maize (ZTDSR–ZT)]. The detail description of different tillage based crop establishment practices are given in Table 2. In residue retention treatment, ~33% of total aboveground crop residue was retained. For that, rice and wheat crops were harvested with a combine at ~30 cm above ground level and ~70 cm maize stalk was retained in field. In the residue removal treatment, residues of all crops were removed. The experiment was laid out in a split–split plot design with three replications, accommodating crop rotation, residue management, and tillage based crop establishment treatments in the main, sub–, and sub–sub plots, respectively. The dimensions of main, sub, and sub–sub plots were 21 m × 32 m, 10.5 m × 32 m, and 10.5 m × 7.5 m, respectively.

**Table 2:** Detail description of tillage based crop establishment treatments (Nandan et al., 2018).

Treatment notation	Treatment description	
	Rice	Wheat/maize
Conventional puddled transplanted rice followed by conventional till wheat/maize (CTTPR–CT)	Two dry–harrowing followed by two wet–tillage (puddling) and one planking was followed by manual transplanting of 25–30 days rice seedling with a row spacing of 20 cm and hill to hill spacing of 15 cm.	Wheat was sown by broadcasting in conventionally tilled plots (2 harrowing +2 tillage +1 planking). Maize was sown by dibbling in conventionally tilled (2 harrowing +2 tillage +1 planking) plots.
Non–puddled transplanted rice followed by zero–till wheat/maize (NPTPR–ZT)	Plots were prepared by dry tillage (two harrowing and planking) but not puddled. Plots were flooded one day before (24 h) transplanting to make soil soft and then 25–30 days old rice seedlings were transplanted in non–puddled soil at 20 cm row spacing and hill to hill spacing of 15 cm.	Zero tillage for wheat and maize. Sowing was done using Zero-till happy–seeder machine. Wheat was sown at 20 cm row spacing and maize at 60 cm row spacing. If there were some pre–established weeds prior to wheat and maize sowing, were killed by applying glyphosate at 1.0 kg a.i. ha^−1^.
Zero–till transplanted rice followed by zero–till wheat/maize (ZTTPR–ZT)	Rice seedlings were directly transplanted under zero–tillage conditions. All the pre–established weeds were killed by applying glyphosate at 1.0 kg ai ha^−1^ about a week before transplanting. The plots were flooded one day before transplanting of the seedling to make the soil soft. Line transplanting was done in flooded plots with row spacing of 20 cm and hill to hill spacing of 15 cm	Same as above
Zero–till direct seeded rice followed by zero–till wheat/maize (ZTDSR–ZT)	Rice was directly sown instead of transplanting in the main field under zero–tillage condition. Pre–established weeds were managed as in ZTTPR. Direct–seeding of rice was done using zero–till seed cum fertilizer drill in zero–till flat plots at 20 cm row spacing. The seedling was done on the same day the nursery sowing was done for transplanted rice treatments.	Same as above

### 2.3 Crop management

In ZTDSR–ZT treatment, rice crop (Arize Tez) was sown directly at a row spacing of 20 cm using a zero–till happy–seeder during the first fortnight of June. The seed rate of rice for ZTDSR was 25 kg ha^−1^. Rice nursery was raised on the same day the rice was sown in ZTDSR. About 25–30 days old rice seedlings were transplanted in CTTPR–CT, NPTPR–ZT, and ZTTPR–ZT treatments following a row spacing of 20 cm and 15 cm hill to hill spacing. In both transplanted and DSR crop, fertilizer nitrogen (N), phosphorus (P) and potassium (K) was applied at 120:46:40 (N: P_2_O_5_: K_2_O) kg ha^−1^ was applied. In transplanted rice, 18% N and full dose of P and K along with zinc sulphate (ZnSO_4_) at 25 kg ha^−1^ were applied as basal. Remaining (82%) dose of N was applied in two equal splits at active tillering and panicle initiation stages. Whereas, in ZTDSR, 18% of N and full dose of P and K along with ZnSO_4_ at 25 kg ha^−1^ were applied as basal, and remaining N (82%) in the was top–dressed in three equal splits at 15 days after sowing (DAS), at active tillering and at panicle initiation stages. The source of fertilizer N, P, and K was urea, diammonium phosphate (DAP) and muriate of potash (MOP), respectively.

Wheat crop (HD 2967) was sown during the second fortnight of November with the help of a zero–till–happy–seeder in NPTPR–ZT, ZTTPR–ZT and ZTDSR–ZT treatments with a row spacing of 22.5 cm. However, wheat seeds were manually broadcasted in CTTPR–CT treatment. The dose of N: P_2_O_5_: K_2_O applied to the wheat crop was 120:60:40 kg ha^−1^. In ZT wheat, 1/5th quantity of required N and full doses of P and K were applied as basal. The remaining quantity of N was applied in three equal splits using urea after the first irrigation (21 DAS) and the following irrigation (50 DAS), and at flowering stage. In conventionally tilled wheat (CTTPR–CT), 33% N was applied as basal and the remaining N was applied in two equal splits at the first irrigation (21 DAS) and at the following irrigation (50 DAS).

Maize (DeKalb 9120) was sown manually by dibbling at a spacing of 60 cm × 15 cm in CTTPR–CT. In NPTPR–ZT, ZTTPR–ZT, and ZTDSR–ZT treatments, maize were sown along with basal fertilizer using a zero–till–happy–seeder (inclined plate seed metering system) with 60 cm row spacing and 15 (±1) cm plant to plant spacing. Fertilizer dose of 150: 75: 50 kg ha^−1^ (N: P_2_O_5_: K_2_O) was applied to the maize crop. The 1/5th quantity of N and full doses of P and K were applied at sowing. The remaining dose of N in the form of urea was applied in three equal split at 30 and 60 DAS, and at the tasseling stage.

### 2.4 Soil sampling and processing

After six years of rotation (at rice harvest in 2015), soil samples were collected from surface soil depth (0–0.2 m) of each plot. In each plot, samples were collected from four random positions and then blended for a representative soil sample. The processed air–dried soil sample (2–mm sieved) was analyzed for soil C–fractions, pH, electrical conductivity (EC) and available nutrients. For aggregate size analysis and aggregate associated C, undisturbed surface soil (0–0.2 m) was sampled using a core sampler. Four cores from each plot were collected. After drying in the shed, the soil was ground by giving gentle strokes with a wooden hammer and aggregates of 4–8 mm size were used for analysis (Majumder et al., 2008).

### 2.5 Analysis of carbon fractions, and computation of C–stabilization

Soil C–fractions were analyzed following the modified Walkley and Black method as described by Chan et al. (2001). Briefly, 5, 10, and 20 ml concentrated H_2_SO_4_ brought about an acid aqueous medium with three proportions of 0.5:1, 1:1, and 2:1, which ultimately led to a solution with the different normality of H_2_SO_4_ i.e. 12, 18 and 24 N, respectively. Here, 20 ml H_2_SO_4_ refers to the original wet oxidation method of Walkley and Black (1934). Briefly, 10 ml 1 N potassium dichromate (K_2_Cr_2_O_7_) solution was used as oxidizer for 1 g soil, and then the mixture was diluted with 200 ml of water. Subsequently, 10 ml H_3_PO_4_ was added. Then excess Cr_2_O_7_^2−^ was titrated with 0.5 N ferrous ammonium sulfate [Fe(NH_4_)_2_(SO_4_)_2_.6H_2_O]. Subsequently, four distinct C–fractions (C*frac*_1_, C*frac*_2_, C*frac*_3,_ and C*frac*_4_) were obtained viz.

Very–labile fraction (C*frac*_1_): The part of organic C oxidized under 12 N H_2_SO_4_.

Labile fraction (C*frac*_2_): Organic C oxidized in 18 N H_2_SO_4_ – Organic C oxidized in 12 N H_2_SO_4_.

Less–labile fraction (C*frac*_3_): Organic C oxidized in 24 N H_2_SO_4_ – Organic C oxidized in 18 N H_2_SO_4_.

Non–labile fraction (C*frac*_4_): Total SOC – Organic C oxidized in 24 N H_2_SO_4._

Finally, for easy interpretation, the sum of very–labile fraction (C*frac*_1_) and labile fraction (C*frac*_2_) was termed active C–pool. While less–labile fraction (C*frac*_*3*_) and non–labile fraction (C*frac*_*4*_) together termed passive C–pool.

Further, the lability index (LI) was derived using very–labile, labile, and less–labile fractions of total SOC, giving a weightage of 3, 2 and 1 to C*frac*_1_, C*frac*_2_, and C*frac*_*3*_, respectively (Blair et al., 1995; Hazra et al., 2018).

(1) $$\mathrm{LI}=\left[\left(\text{very labile}\;C/\mathrm{TOC}\right)\times 3+\left(\text{labile}\;C/\mathrm{TOC}\right)\times 2+\left(\text{less labile}\;C/\mathrm{TOC}\right)\times 1\right]\;$$

Then, the carbon pool index (CPI) was derived as:

(2) $$\mathrm{CPI}=\text{Sample}\;\mathrm{TOC}\;\left(g\;{\mathrm{kg}}^{-1}\right)/\text{Reference}\;\mathrm{TOC}\;\left(g\;{\mathrm{kg}}^{-1}\right)$$

where, conventional cropping practice (CTTPR–CT without crop residue retention) was taken as reference.

Finally, the carbon management index (CMI) was derived using the following formula:

(3) CMI=CPI×LI×100

The total amount of season–wise crop residue applied for the last six years of the respective treatments was measured using a 1 m × 1 m quadrate in each plot. The amount of C added through crop residue was quantified taking after the presumption that 40% of the retained crop residue was C (Yang and Wander, 1999).

(4) $$\mathrm{Carbon stabilized in active}\;C\;\mathrm{pool}\;\left(\%\right)=\frac{\text{active}\;C\;\text{pool in residue retention plot}-\text{active}\;C\;\text{pool in residue removal plot}}{\text{Total residue}\;C\;\text{input}\;}$$

(5) $$\mathrm{Carbon stabilized in passive}\;C\;\mathrm{pool}\;\left(\%\right)=\frac{\text{passive}\;C\;\text{pool in residue retention plot}-\text{passive}\;C\;\text{pool in residue removal plot}}{\text{Total residue}\;C\;\text{input}\;}$$

### 2.6 Analysis of aggregate size class and aggregate associated C

The soil aggregate size classes were determined by the wet sieving method using a Yoder's apparatus (Yoder, 1936). For this purpose, 100 g soil aggregates (4–8 mm size) were shaken in water in a drum for a period of 2 min (approximately 3 cm up and down with the frequency of 50 times) and passed through a series of four sieves (2, 0.5, 0.25 and 0.053 mm). The soil material and water passing through the sieves were poured onto a smaller mesh sieve (53-mm sieve) and the sieving procedure was repeated. The four aggregate size classes namely, coarse macro–aggregates (>2.0 mm), meso–aggregates (0.25–2.0 mm), coarse micro–aggregates (0.053–0.25 mm) and ‘silt + clay’ sized fraction (<0.053 mm) were obtained. Aggregate fractions retained on each sieve were transferred into a container and dried at 65 °C. Accordingly, water stable macro–aggregate (WSMacA, >0.25 mm) and micro–aggregate (WSMicA, <0.25 mm) were calculated and their ratio was designated as aggregate ratio (AR) (Oades and Waters, 1991).

Aggregate class was then separated into and their ratio was designated as aggregate ratio (AR) (Oades and Waters, 1991). Aggregate mean weight diameter (MWD) was determined by the following equation (Kemper and Rosenau (1986):

(6) $$\mathrm{MWD}\;\left(\mathrm{mm}\right)=\sum X_{i}\times W_{i}$$

where, W_i_ is the proportion of aggregates retained in the sieves in relation to the whole, X_i_ is the mean diameter of the size class (mm).

The cumulative values of organic C present in soil aggregate of >2.0 mm, 0.25–2.0 mm, 0.053–0.25 mm, and <0.053 mm were considered coarse macro–aggregated C (CMacAC), meso–aggregated C (MesAC), coarse micro–aggregated C (CMicAC) and ‘silt+clay’ associated C, respectively (Bandyopadhyay et al., 2010). Likewise, the organic C content in macro–aggregate and micro–aggregates were designated macro–aggregated C (MacAC) and micro–aggregated C (MicAC), respectively. The soil of each aggregate size class was first treated with HCl to make soils free from inorganic C, and then TOC was estimated using TOC analyzer (Analytikjena Multi N/C analyzer, Model 2100).

### 2.7 Soil chemical analysis

The soils were analyzed for available–N (Alkaline KMnO_4_ method), available–P (Olsen's extractant, 0.5 N NaHCO_3_, pH 8.5), available–K (1 N NH_4_OAc extractable K, pH 7.0), and available–S (0.01 M CaCl_2_ extractable) following the standard methods. The DTPA extractable–Zn was estimated using an Atomic Absorption Spectrometer (Lindsay and Norvell, 1978). Soil pH and EC were estimated using the methods depicted by Jackson (1973).

### 2.8 Statistical analysis

The data were analyzed using online OPSTAT statistical program (Sheoran et al., 1998). The significance of ‘F’ value was determined based on analysis of variance (ANOVA) for split–split plot design. Least significant difference (LSD) at *p* = 0.05 was used for multiple comparisons of treatment means. For Pearson correlation matrix, Excel based data analysis Tool Pack was used. The principal component analysis was performed using the PAST (3.14) software.

## 3 Results

### 3.1 Carbon fractions and C–stabilization

**Fig. 1:**
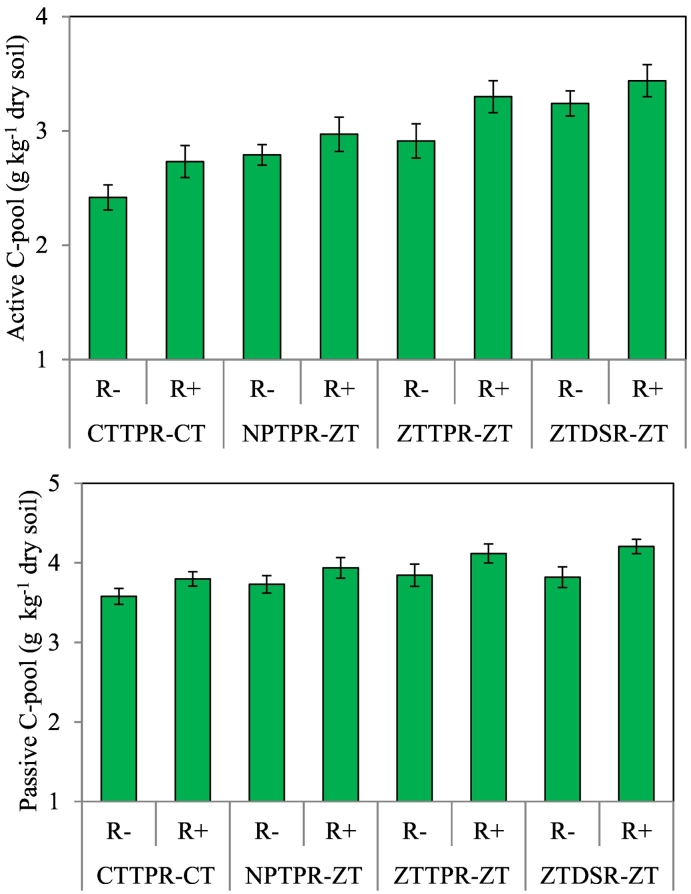
Effect of tillage based crop establishment practices and residue management on active and passive C-pool after six years of crop rotation. *CTTPR-CT*, puddled transplant rice followed by conventional till wheat/maize; *NPTPR-ZT*, non-puddled transplant rice followed by zero-till wheat/maize; *ZTTPR-ZT*, zero-till transplant rice followed by zero-till wheat/maize; *ZTDSR-ZT*, zero-till direct seeded rice followed by zero-till wheat/maize; R-, residue removal; R+, residue retention. Error bar represents standard error of mean.

Tillage based crop establishment practices and residue management treatments strongly influenced TOC and soil C–fractions, C–pools, and C–management indices (Table 3 and Fig. 1). Residue retention treatment increased C*frac*_1*,*_ C*frac*_2*,*_ C*frac*_3*,*_ C*frac*_4*,*_ and TOC by 18, 24, 5, 10, and 12%, respectively, over residue removal treatment. Conservation tillage treatments (NPTPR–ZT, ZTTPR–ZT and ZTDSR–ZT) had 13–21%, 12–16%, 5–7%, 9–13%, and 9–14% higher (*p* < 0.05) C*frac*_1_, C*frac*_2_, C*frac*_3_, C*frac*_4_, and TOC, respectively, over CTTPR–CT. ZTDSR–ZT and ZTTPR–ZT treatments increased (*p* < 0.05), active C–pool, LI and CMI over CTTPR–CT. Notably, in the study, the C*frac*_3_ (32% of TOC) was the dominant C–fraction, followed by C*frac*_1_ (31%), C*frac*_4_ (25%), C*frac*_2_ (12%).

**Table 3:** Total organic carbon (TOC), soil carbon fractions and carbon indices as influenced by different cropping system, residue management and tillage based crop establishment treatments.

Treatment	Carbon fractions (g kg^−1^)				TOC (g kg^−1^)	AP: PP	LI	CMI
	C*frac*_1_	C*frac*_2_	C*frac*_3_	C*frac*_4_				
Cropping system								
Rice–wheat	2.13	0.87	2.20	1.69	6.89	0.77	1.50	112.7
Rice–maize	2.19	0.83	2.27	1.73	7.02	0.76	1.50	109.7
LSD (*p* = 0.05)	ns	ns	ns	ns	ns	ns	ns	ns

Residue management								
Residue removal	1.98	0.76	2.18	1.62	6.55	0.72	1.47	108.4
Residue retention	2.34	0.94	2.29	1.79	7.36	0.80	1.52	114.0
LSD (*p* = 0.05)	0.07	0.03	0.07	0.05	0.14	0.02	0.03	5.3

Tillage based crop establishment practice								
CTPTR–CT	1.90	0.77	2.14^b^	1.57	6.38	0.72	1.47	100.0
NPTPR–ZT	2.15	0.86	2.24^a^	1.71	6.96	0.76	1.49	111.2
ZTTPR–ZT	2.30	0.89	2.27^a^	1.78	7.25	0.79	1.51	117.0
ZTDSR–ZT	2.29	0.88	2.29^a^	1.78	7.24	0.78	1.51	116.6
LSD (*p* = 0.05)	0.06	0.04	0.05	0.07	0.12	0.03	0.03	5.9

C*frac*_1_, very–labile C fraction; C*frac*_2_, labile C fraction; C*frac*_3_, less–labile C fraction; C*frac*_4_, non–labile C fraction; *AP: PP*, active C–pool: passive C–pool; *LI*, lability index; *CMI*, carbon management index.

**Fig. 2:**
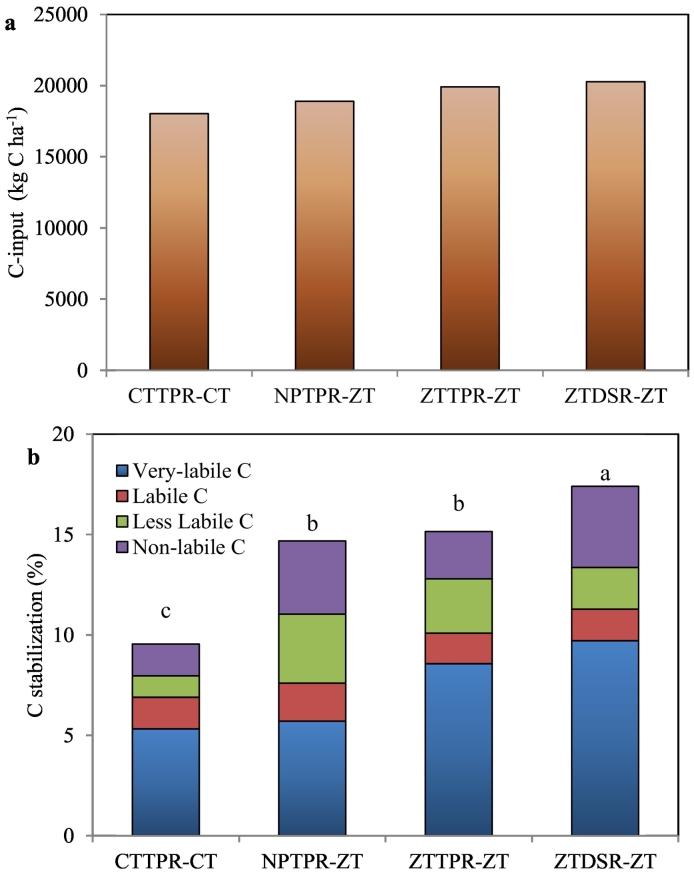
Cumulative C-input through crops residue (a) and C-stabilization in different C-fractions (b) as influenced by different tillage based crop establishment practices in rice-based cropping systems. *CTTPR-CT*, puddled transplant rice followed by conventional till wheat/maize; *NPTPR-ZT*, non-puddled transplant rice followed by zero-till wheat/maize; *ZTTPR-ZT*, zero-till transplant rice followed by zero-till wheat/maize; *ZTDSR-ZT*, zero-till direct seeded rice followed by zero-till wheat/maize. The columns with different letters are significantly different at *p* ≤ 0.05.

A strong integrated effect of conservation tillage (zero–tillage/reduced tillage) with crop residue retention over conventional CTTPR–CT without residue retention was observed on soil quality parameters. Irrespective of the cropping system, ZTDSR–ZT or ZTTPR–ZT with crop residue retention had 29–30% higher TOC over conventional CTTPR–CT without residue retention (Table 3). Stabilization of added carbon in soil was the highest in ZTDSR–ZT and reduced progressively to the order of ZTDSR–ZT > ZTTPR–ZT ≥ NPTPR–ZT > CTTPR–CT (Fig. 2b). ZT based crop establishment treatments increased stabilization of C*frac*_1_, C*frac*_3_, and C*frac*_4_ over CTTPR–CT.

### 3.2 Soil aggregates and aggregate associated C

Residue management and tillage based crop management practices significantly influenced the distribution of soil aggregates size fraction, MWD, and AR but cropping system did not influence these parameters (Table 4). Residue retention treatment increased (*p* < 0.05) the content of coarse macro–aggregate and meso–aggregate over residue removal treatment. The ZT based crop establishment treatments (ZTTPR–ZT and ZTDSR–ZT) had higher content of coarse macro–aggregate and meso–aggregate over CTTPR–CT. Subsequently, the MWD and aggregate ratio (AR) were higher in residue retention and zero–till crop establishment treatments. Retention of crop residue increased the WSMacA by 7% over residue removal treatment. The ZT based treatments ZTTPR–ZT, ZTDSR–ZT, and NTTPR–ZT increased WSMacA by 16, 15, and 9%, respectively over CTTPR–CT.

**Table 4:** Effect of cropping system, residue management and tillage based crop establishment treatments on soil aggregate size distribution, mean weight diameter (MWD), and aggregate ratio (AR) after six years of crop rotation.

Treatment	Percent share of aggregate size class (%)				WSMacA (%)	WSMicA (%)	MWD (mm)	AR
	>2 mm	0.25–2 mm	0.053–0.25 mm	<0.053 mm				
Cropping system								
Rice–wheat	33.1	45.0	9.7	12.2	78.1	21.9	1.52	4.02
Rice–maize	34.3	46.9	8.4	10.4	81.2	18.8	1.57	4.66
LSD (*p* = 0.05)	ns	ns	ns	ns	ns	ns	ns	ns

Residue management								
Residue removal	32.4	44.5	10.0	13.1	76.9	23.1	1.49	3.57
Residue retention	35.0	47.3	8.2	9.5	82.3	17.7	1.60	5.12
LSD (*p* = 0.05)	2.1	2.4	ns	1.5	2.38	2.4	0.06	1.14

Tillage based crop establishment practice								
CTPTR–CT	30.5	42.0	14.6	12.9	72.5	27.5	1.41	2.73
NPTPR–ZT	34.2	44.7	8.6	12.5	79.0	21.1	1.55	4.00
ZTTPR–ZT	34.9	48.9	6.1	10.1	83.8	16.2	1.61	5.58
ZTDSR–ZT	35.2	48.0	7.0	9.8	83.2	16.8	1.61	5.06
LSD (*p* = 0.05)	3.6	3.1	3.1	1.9	3.5	3.48	0.06	1.6

*WSMacA*, water stable macro-aggregates; *WSMicA*, water stable micro-aggregates.

**Fig. 3:**
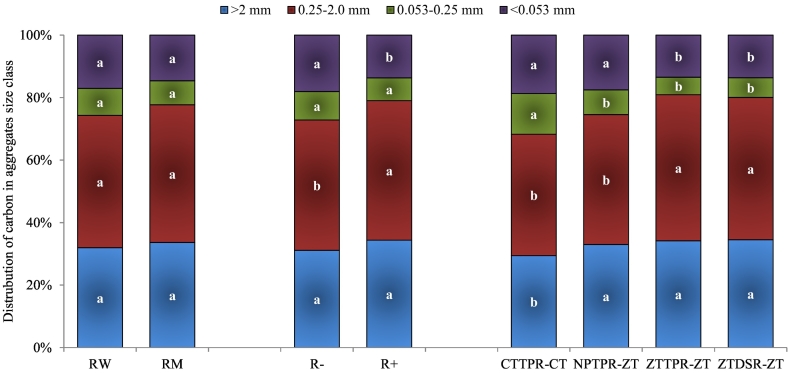
Allocation of C (%) in different aggregates size classes (>2, 0.25–2 0.0, 0.053–0.25, <0.053 mm) of surface soil (0–0.2 m) as influenced by crop rotation, residue retention, and tillage based crop establishment practices after six-year of rotation. *RW*, rice-wheat; *RM*, rice-maize; *R−*, residue removal; *R+*, residue retention; *CTTPR-CT*, puddled transplant rice followed by conventional till wheat/maize; *NPTPR-ZT*, non-puddled transplant rice followed by zero-till wheat/maize; *ZTTPR-ZT*, zero-till transplant rice followed by zero-till wheat/maize; *ZTDSR-ZT*, zero-till direct seeded rice followed by zero-till wheat/maize. Different lowercase letters in the same colour columns (for each main factor) are significantly different at *p* ≤ 0.05.

**Table 5:** Effect of cropping system, residue management and tillage based crop establishment treatments on soil aggregate associated carbon concentration.

Treatment	Aggregate associated carbon (g kg^−1^)			
	CMacAC	MesAC	CMicAC	Silt + clay
Cropping system				
Rice–wheat	8.45	8.24	7.97	12.28
Rice–maize	8.72	8.37	8.17	12.59
LSD (*p* = 0.05)	0.07	0.11	ns	ns

Residue management				
Residue removal	8.25	8.05	7.96	11.85
Residue retention	8.92	8.56	8.18	13.03
LSD (*p* = 0.05)	0.35	0.19	0.21	0.64

Tillage based crop establishment practice				
CTPTR–CT	8.31	7.96	7.77	12.60
NPTPR–ZT	8.57	8.28	8.19	12.65
ZTTPR–ZT	8.66	8.48	8.15	11.94
ZTDSR–ZT	8.80	8.51	8.16	12.56
LSD (*p* = 0.05)	0.15	0.22	0.23	0.56

*CMacAC*, coarse macro–aggregated carbon; *MesAC*, meso–aggregated carbon; *CMicAC*, coarse micro–aggregated carbon. *ns*, non-significant (*p* > 0.05).

The content of organic carbon in different aggregate size classes followed the order: ‘silt + clay’ C > coarse macro–aggregated C (CMacAC) > meso–aggregated C (MesAC) > coarse micro–aggregated C (CMicAC) (Table 5). Residue retention treatment had higher (*p* < 0.05) organic carbon content in all aggregate size classes, being higher in ‘silt + clay’ (10%) followed by macro–aggregate (8%) and meso–aggregate (6%) and was least in micro–aggregates (3%). Zero tillage based crop establishment treatments (ZTTPR–ZT, ZTDSR–ZT) resulted in higher concentration of organic carbon in meso–aggregate and coarse macro–aggregate, but had no influence on organic carbon content in ‘silt + clay’. Fig. 3 illustrates that the percent share of CMacAC and MesAC increased in ZT based crop establishment treatments, while CMicAC and ‘silt+clay’ C decreased compared to CTTPR–CT. Notable, the interaction of residue management × tillage based crop establishment treatment and cropping system × tillage based crop establishment treatment was significant (*p* < 0.05) for MesAC and CMicAC (Supplementary Table 1).

### 3.3 Soil chemical properties

At the end of six years, residue retention treatment increased (*p* < 0.05) soil available N, P, K, S, and DTPA–extractable Zn by ~10, 16, 12, 6, and 11%, respectively, over residue removal treatment (Table 6). However, no specific trend was noticed in tillage based crop establishment treatments for soil available nutrients. Crop rotation effect was also non–significant on soil available nutrients. Notably, the interaction residue management × tillage based crop establishment treatments was found significant for available–P, available––K and DTPA extractable Zn (Suppl. Table 1).

**Table 6:** Effect of crop rotation, residue retention, and tillage based crop establishment treatments on soil chemical properties and available nutrients after six years crop rotation.

Treatment	pH	EC	Available–N (kg ha^−1^)	Available–P (kg ha^−1^)	Available–K (kg ha^−1^)	Available–S (kg ha^−1^)	DTPA extractable Zn (mg kg^−1^)
Cropping system							
Rice–wheat	7.39	0.74	188.1	29.3	242.8	11.63	0.86
Rice–maize	7.46	0.66	186.6	27.7	232.1	11.94	0.85
LSD (*p* = 0.05)	ns	ns	ns	ns	ns	ns	ns

Residue management							
Residue removal	7.48	0.73	178.7	26.4	224.2	11.42	0.81
Residue retention	7.38	0.66	195.9	30.6	250.6	12.15	0.90
LSD (*p* = 0.05)	0.07	ns	4.91	2.5	19.3	0.55	0.05

Tillage based crop establishment practice							
CTPTR–CT	7.44	0.63	185.8	29.0	236.2	11.62	0.84
NPTPR–ZT	7.48	0.62	182.9	29.0	226.8	12.03	0.92
ZTTPR–ZT	7.41	0.75	185.0	27.5	222.4	11.47	0.88
ZTDSR–ZT	7.39	0.79	195.5	28.4	264.3	12.01	0.79
LSD (*p* = 0.05)	ns	ns	13.1	ns	20.9	ns	0.06

*ns*, non-significant (*p* > 0.05).

### 3.4 Correlations and grain yield

**Fig. 4:**
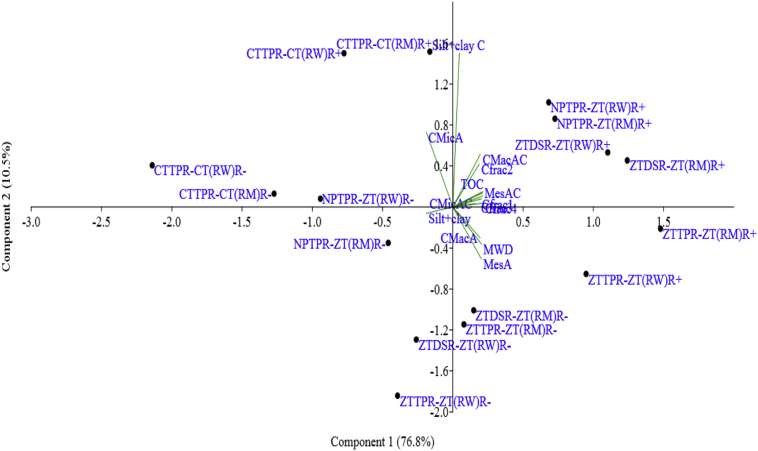
Principal component analysis (PCA) of soil variables for treatment combination of cropping system, residue management and tillage and crop establishment treatments. *RW*, rice-wheat; *RM*, rice-maize; *R−*, residue removal; *R+*, residue retention; *CTTPR-CT*, puddled transplant rice followed by conventional till wheat/maize; *NPTPR-ZT*, non-puddled transplant rice followed by zero-till wheat/maize; *ZTTPR-ZT*, zero-till transplant rice followed by zero-till wheat/maize; *ZTDSR-ZT*, zero-till direct seeded rice followed by zero-till wheat/maize. Coarse macro-aggregated (CMacA), meso-aggregated (MesA), coarse micro-aggregated *CMacA*, coarse macroaggregates; *MesA*, mesoaggregates; *CMicA*, coarse microaggregates; *MWD*, mean weight diameter; *CMacAC*, coarse macro–aggregated carbon; *MesAC*, meso–aggregated carbon; *CMicAC*, coarse micro–aggregated carbon; C*frac*_1_, very–labile carbon fraction; C*frac*_2_, labile carbon fraction; C*frac*_3_, less–labile carbon fraction; C*frac*_4_, non–labile carbon fraction; *TOC*, total organic carbon.

Strong association between soil aggregate size classes, MWD, aggregate–associated C content, and C–fractions were observed at sixth year of rotation (Table 7). The PCA graph shows that the combination of zero-tillage based crop establishment with residue retention (positioned on the right side of PCA coordinates) had strong impact on the aggregation and soil carbon parameters (Fig. 4). The improvement in soil properties with conservation tillage based crop establishment practices and crop residue retention strongly influenced the crop productivity (Table 8). Significantly higher rice grain yield was recorded in ZTDSR–ZT treatment than other tillage based crop establishment treatments, where the rice grain yield in NPTPR–ZT and CTTPR–CT treatments were comparable. The ZT–based crop establishment practices had higher wheat and maize grain yields than CTTPR–CT. Residue retention increased productivity of all the crops, being the highest positive on maize yield (7–10%), followed by wheat (5–11%) and rice (3–8%).

**Table 7:** Pearson correlation matrix soil variables with response to crop rotation, residue retention, and tillage based crop establishment treatments.

	CMacA	MesA	CMicA	Silt+clay	MWD	CMacAC	MesAC	CMicAC	Silt+clay C	C*frac*_1_	C*frac*_2_	C*frac*_3_	C*frac*_4_	TOC
CMacA	1.00													
MesA	0.76⁎⁎	1.00												
CMicA	−0.80⁎⁎	−0.83⁎⁎	1.00											
Silt+clay	−0.76⁎⁎	−0.77⁎⁎	0.59⁎	1.00										
MWD	0.90⁎⁎	0.83⁎⁎	−0.83⁎⁎	−0.80⁎⁎	1.00									
CMacAC	0.62⁎	0.69⁎	−0.55⁎	−0.73⁎⁎	0.67⁎	1.00								
MesAC	0.73⁎⁎	0.80⁎⁎	−0.71⁎⁎	−0.76⁎⁎	0.78⁎⁎	0.80⁎⁎	1.00							
CMicAC	0.49	0.60⁎	−0.61⁎	−0.41	0.54⁎	0.64⁎	0.64⁎	1.00						
Silt+clay C	0.10	−0.11	0.20	−0.23	0.05	0.42	0.23	0.16	1.00					
C*frac*_1_	0.74⁎⁎	0.79⁎⁎	−0.73⁎⁎	−0.73⁎⁎	0.78⁎⁎	0.80⁎⁎	0.86⁎⁎	0.57⁎	0.20	1.00				
C*frac*_2_	0.60⁎	0.62⁎	−0.57⁎	−0.60⁎	0.63⁎	0.72⁎⁎	0.80⁎⁎	0.47	0.32	0.84⁎⁎	1.00			
C*frac*_3_	0.73⁎⁎	0.77⁎⁎	−0.71⁎⁎	−0.72⁎⁎	0.77⁎⁎	0.79⁎⁎	0.81⁎⁎	0.70⁎	0.23	0.77⁎⁎	0.65	1.00		
C*frac*_4_	0.75⁎⁎	0.80⁎⁎	−0.76⁎⁎	−0.71⁎⁎	0.79⁎⁎	0.79⁎⁎	0.87⁎⁎	0.62⁎	0.17	0.90⁎⁎	0.84⁎⁎	0.81⁎⁎	1.00	
TOC	0.74⁎⁎	0.78⁎⁎	−0.73⁎⁎	−0.72⁎⁎	0.78⁎⁎	0.81⁎⁎	0.87⁎⁎	0.60⁎	0.23	0.90⁎⁎	0.85⁎⁎	0.81⁎⁎	0.91⁎⁎	1

*CMacA*, coarse macroaggregates; *MesA*, mesoaggregates; *CMicA*, coarse microaggregates; *MWD*, mean weight diameter; *CMacAC*, coarse macro–aggregated carbon; *MesAC*, meso–aggregated carbon; *CMicAC*, coarse micro–aggregated carbon; C*frac*_1_, very–labile carbon fraction; C*frac*_2_, labile carbon fraction; C*frac*_3_, less–labile carbon fraction; C*frac*_4_, non–labile carbon fraction; *TOC*, total organic carbon.

⁎ *p* < 0.05, two–tailed.

⁎⁎ *p* < 0.01, two–tailed.

**Table 8:** Rice, wheat and maize grain yield in 2013–2014 and 2014–2015 under different cropping systems, residue management and tillage based crop establishment treatments (Nandan et al., 2018).

Treatment	Rice (kg ha^−1^)		Wheat (kg ha^−1^)		Maize (kg ha^−1^)	
	2013–14	2014–15	2013–14	2014–15	2013–14	2014–15
Cropping system						
Rice–wheat	4846	4579				
Rice–maize	4752	4532				
LSD (*p* = 0.05)	ns	ns				

Residue management						
Residue removal	4609	4489	5022	5117	6903	6852
Residue retention	4989	4623	5288	5657	7617	7323
LSD (*p* = 0.05))	122	131	158	160	256	239

Tillage based crop establishment practice						
CTPTR–CT	4486	4194	4586	4676	6756	6669
NPTPR–ZT	4602	4190	5071	5512	7168	6914
ZTTPR–ZT	4875	4612	5341	5625	7535	7343
ZTDSR–ZT	5232	5226	5621	5734	7580	7424
LSD (*p* = 0.05)	224	229	409	396	417	249

*ns*, non-significant (*p* > 0.05).

## 4 Discussion

### 4.1 Carbon fractions and C–stabilization

Minimizing soil oxidation remains crucial for carbon sequestration in tropical soils. The increased TOC in zero–tillage/reduced tillage is possibly because of minimum mechanical disturbance of soil and restricted of soil carbon oxidation. Intensive tillage practices accelerate soil organic matter (SOM) mineralization (Elder and Lal, 2008). Very–labile C–fraction (C*frac*_1_) and labile C–fraction (C*frac*_2_) are highly prone to oxidation processes (Nath et al., 2017a). Therefore, higher concentrations of C*frac*_1_ and C*frac*_2_ in zero–till based crop establishment treatments indicate that restricted oxidation of organic carbon in conservation tillage treatments. Results further suggests that elimination of tillage could increase recalcitrant C–pool as the higher content of less–labile C–fraction (C*frac*_3_) and non–labile C–fraction (C*frac*_4_) was observed in zero–till crop establishment treatments. The results exclusively demonstrate that even a single wet tillage (puddling) operation could result in substantial SOC loss and that may be the reason for less impact of NPTPR–ZT than ZTTPR–ZT and ZTDSR–ZT. Increased TOC in residue retention treatment was mainly because of increased addition of C–input. This way, conservation tillage based crop establishment in combination with residue retention may lead to a strong positive impact on soil health, particularly on SOC level. Thus, the results demonstrate present relevance of conservation agriculture in tropical rice–based production system for restoration of soil health particularly SOC in surface soil (0–0.2 m) which is crucial for crop production.

Fundamentally, the reduced oxidation processes in lowland flooded rice soil increase accumulation of less–labile and non–labile C–fractions (Jenkinson, 1988). Majumder et al. (2008) and Benbi et al. (2012) reported that *Cfrac*_3_ and *Cfrac*_4_ together constituted the major share of TOC in rice soils of hot humid tropics. In anaerobic rice soil, slow mineralization rate of C substrates resulted in cumulative accumulation of resistant and less oxidizable C compounds (e.g. lignins) (Singh et al., 2005). In the present study, the significant variation in C*frac*_4_ was observed with crop residue and tillage based crop establishment treatments. These results are in contrast to the findings of Blair et al. (1995), who found that non–labile (C*frac*_4_) are mostly non–sensitive to crop and soil management (Table 3). This contradiction might be specific to rice ecology, which facilitates faster conversion of C–input to resistant C–pool.

In the study, conservation tillage treatments (NTTPR–ZT, ZTTPR–ZT and ZTDSR–ZT) increased the C stabilization in passive pool (C*frac*_3_ and C*frac*_4_), which indicates that retention of crop residue with conservation tillage practice could increase passive C–pool in tropical rice soils. According to Ghosh et al. (2016) the higher content of less–decomposable lignin and cellulose in cereal residue is effective for improving recalcitrant C–pool that persists longer in soils.

### 4.2 Aggregate and aggregate–associated carbon and soil fertility

Aggregate stability and proportion of macro–aggregate strongly influence carbon sequestration, and often degradation of large aggregates induces SOC loss (Lal, 1997). C–stabilization is strongly associated with aggregate size composition (Andruschkewitsch et al., 2014b). Intensive tillage practices cause physical disruption of macro–aggregates and expose SOM to microbial decomposition (Six et al., 2000; Zotarelli et al., 2007). According to Doran (1980) soil microbial and biochemical environment of zero–till soils is less oxidative than that under conventional tillage. In consistent with previous findings, the higher water stable macro–aggregates were observed in conservation tillage treatments, especially in zero tillage treatments (ZTTPR–ZT and ZTDSR–ZT). Plant roots and rhizosphere also influences soil aggregation (Bronick and Lal, 2005). Puddling in rice season develops soil compaction that largely restricts root growth of succeeding crop and this might have negative impact on soil aggregation. A strong positive relationship between SOC and the proportion of macro–aggregates has been reported by many researchers (Spohn and Giani, 2010; Huang et al., 2010). Besides this, the release of polysaccharide compounds during the decomposition of crop residue acts as a cementing agent and has a crucial role in macro–aggregate formation (Bandyopadhyay et al., 2010; Choudhury et al., 2014). In this study, the effect of crop residue retention was likewise prominent and significant on soil aggregation.

System based conservation tillage treatments increased C concentration in CMacA, MesA, and CMicA over CTTPR–CT treatment. Kumari et al. (2011) also observed higher macro–aggregated carbon in ZT based crop establishment practices compared with conventional tillage practice in a rice–wheat cropping system. The average carbon concentration within aggregates was of the order: CMacAC > MesAC > CMicAC, indicating that the increased carbon density was proportional to aggregates size (Tisdall and Oades, 1982). According to the conceptual model of Tisdall and Oades (1982) SOM associated with macro–aggregates is less persistent than that of micro–aggregates. On the same line, Six et al. (2002) reported that the carbon content in silt+clay fraction represents the most strongly protected C in soils. In our study, residue retention treatments improved the ‘silt + clay’ carbon by ~10% (*p* < 0.05) in 6 years. Likewise, higher C concentration in CMicA under conservation tillage treatments has special significance because of its longer persistence in the soil.

The improved soil fertility with retention of crop residues was mainly because of additional nutrient input through left over crop residue in addition to the recommended mineral fertilizers. The effect of crop residue on soil available nutrients are expected to be additive over time. Notably, increased SOC often associates with higher microbial activity that also directly influences availability of P and S in the soil. The hydrolysis of organic materials results in low molecular weight aliphatic acids (LOAs). The competitive sorption between these low molecular weight acids and P for soil sorption sites results in an increasing concentration of solution P (Guppy et al., 2005). Cereal crop residues are known as the rich source of K and thus retention of crop residues increased K in the soil. The results further suggest that, in long–term, the dose of mineral fertilizers may be reduced in residue retention treatments. Despite increased crop productivity in ZTDSR–ZT and ZTTPR–ZT over CTTPR–CT, the soil fertility was comparable to CTTPR–CT. This indicates that conservation tillage practices did not have any adverse impact on soil nutrient availability and the current mineral fertilizer rate is adequate.

### 4.3 Correlations and grain yield

**Fig. 5:**
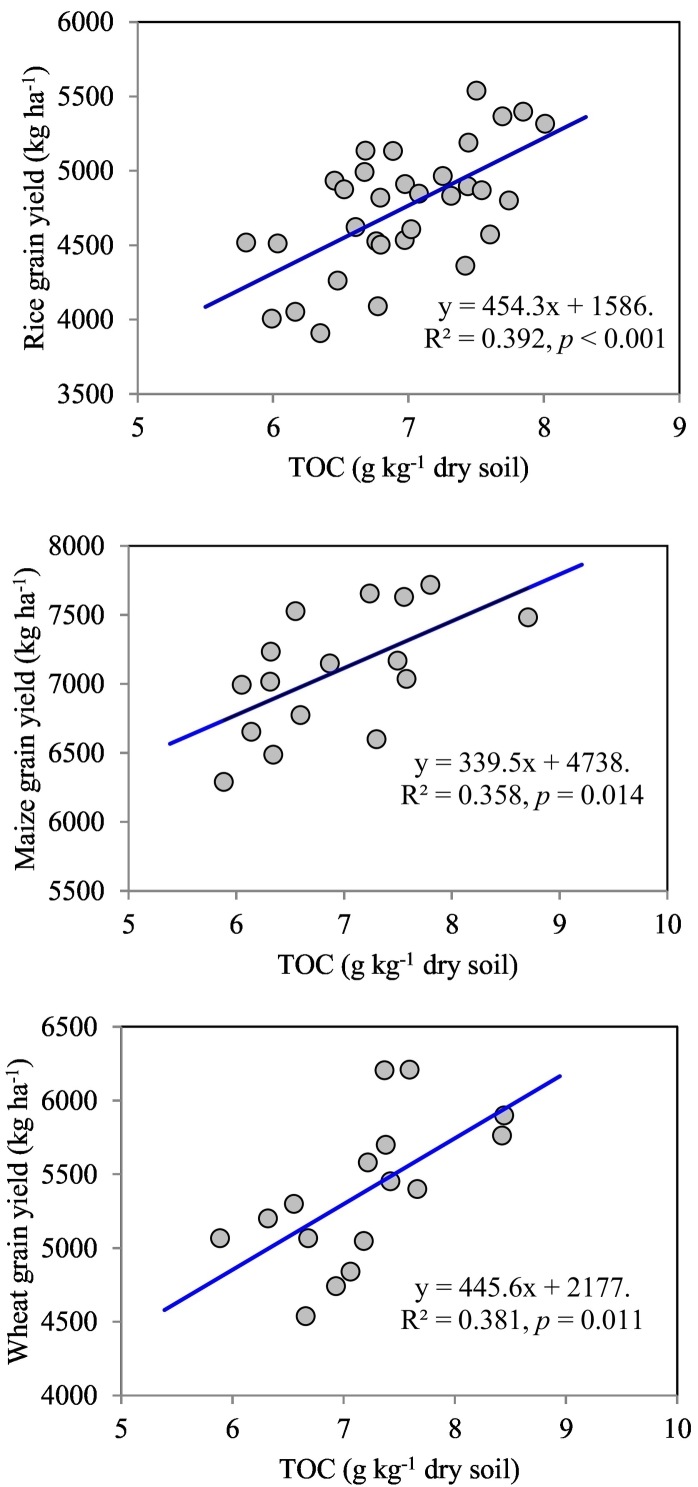
Relationship between total organic carbon (TOC) (g kg^−1^ dry soil) and grain yield of rice, maize, and wheat crop (kg ha^−1^) at six years of crop rotation.

The strong relationship between TOC and grain yield of rice (*r* = 0.63, *p* < 0.001), wheat (*r* = 62, *p* < 0.05), and maize (*r* = 0.60, *p* < 0.05) (Fig. 5) reflects the importance of SOC in sustaining the crop productivity of rice–based cropping system of the tropical IGP. The higher response of winter crops (wheat and maize) to crop residue retention and ZT (wheat only) might be associated with the higher conserved soil moisture, improvement in physical properties, and moisture dependent plant nutrient accessibility. Higher wheat yield under ZT (with and without residue retention) compared with conventional tillage in eastern IGP region have also been reported in similar studies (Laik et al., 2014; Keil et al., 2015; Nath et al., 2017b). Likewise, Gathala et al. (2011) observed 9–10% higher yield under ZT combined with residue mulch compared to the conventional tillage and ZT without crop residue.

## 5 Conclusions

It is concluded that zero–till crop establishment practices (ZTTPR–ZT and ZTDSR–ZT) in rice–based systems had a positive impact on soil organic C–pools, macro–aggregate formation, and carbon stock in aggregates. Conservation tillage treatments increased the stabilization of residue C–input compared to conventional CTTPR–CT. Our results suggest that conservation tillage treatments in rice–based cropping systems could maintain higher passive C–pool over CTTPR–CT and thus upgrade the quality of organic carbon, which persist longer in the soil. The effect of crop residue retention on TOC and soil available nutrients was very prominent at the end of six-year rotation. The effect was highest for available–P, followed by available–K, and DTPA–extractable Zn. Strong positive correlations between TOC and component crops productivity were also observed. Thus, embracing resource conserving or conservation tillage–based crop establishment practices combined with residue retention are crucial for efficient soil C management and for sustainability of the rice–based systems in the region.

The following is the supplementary data related to this article.

Supplementary Table 1 *p* value of treatment main effects and their interactions for different soil parameters. Supplementary Table 1
