# Supplementary material for: Impact of conservation tillage in rice–based cropping systems on soil aggregation, carbon pools and nutrients
Source: Geoderma. 2019 Apr 15;340:104–14. doi: 10.1016/j.geoderma.2019.01.001 (PMC6358044; doi:10.1016/j.geoderma.2019.01.001)
Supplement: Supplementary Table 1 — p value of treatment main effects and their interactions for different soil parameters. [file mmc1.docx]

**Supplementary Table 1.**

*p* value of treatment main effects and their interactions for different soil parameters.

|  | *p* value | | | | | | |
| --- | --- | --- | --- | --- | --- | --- | --- |
|  | Main effect | | | Interactions | | | |
| Soil parameter | Cropping system (C) | Residue management  (R) | Tillage based crop establishment practice  (TCE) | C×R | C×TCE | R×TCE | C×R× TCE |
| C*frac_1_* | 0.124 | **<0.001** | **<0.001** | 0.144 | **<0.001** | 0.475 | 0.335 |
| C*frac_2_* | 0.339 | **<0.001** | **<0.001** | 0.061 | 0.070 | 0.753 | 0.071 |
| C*frac_3_* | 0.111 | 0.031 | **0.011** | 0.113 | 0.059 | 0.557 | 0.163 |
| C*frac_4_* | 0.116 | **<0.001** | **0.007** | 0.279 | **<0.001** | 0.525 | 0.368 |
| TOC | 0.132 | **<0.001** | **<0.001** | 0.200 | **<0.001** | 0.414 | 0.469 |
| LI | 0.210 | **0.037** | **0.019** | 0.020 | 0.000 | 0.562 | 0.162 |
| CMI | 0.071 | 0.021 | **<0.001** | 0.953 | **<0.001** | 0.632 | 0.406 |
| pH | 0.070 | **0.039** | **0.069** | 0.078 | 0.079 | 0.129 | 0.070 |
| EC | 0.134 | 0.387 | 0.079 | 0.365 | 0.190 | 0.985 | 0.059 |
| AN | 0.174 | **<0.001** | 0.043 | 0.249 | 0.117 | 0.079 | **0.043** |
| AP | 0.119 | **<0.001** | 0.075 | **0.009** | 0.076 | **0.019** | **0.045** |
| AK | 0.082 | **0.031** | **0.005** | **0.013** | **0.008** | **0.001** | **0.007** |
| AS | 0.253 | **0.013** | 0.074 | 0.263 | 0.122 | 0.137 | 0.085 |
| DTPA-Zn | 0.155 | **0.017** | **<0.001** | 0.074 | **0.011** | **0.041** | **0.009** |
| CMacA | 0.282 | **0.033** | 0.027 | 0.401 | **0.045** | **0.003** | 0.119 |
| MesA | 0.072 | **0.009** | **<0.001** | **0.044** | 0.738 | 0.709 | 0.895 |
| CMicA | 0.099 | 0.054 | **<0.001** | 0.419 | 0.526 | 0.561 | 0.817 |
| Silt+clay | 0.072 | **<0.001** | **<0.001** | 0.274 | **0.043** | 0.247 | 0.051 |
| WSMacA | 0.098 | **<0.001** | **<0.001** | 0.918 | 0.071 | 0.060 | 0.150 |
| WSMicA | 0.053 | **<0.001** | **<0.001** | 0.114 | 0.057 | 0.179 | 0.489 |
| CMacAC | **0.001** | **<0.001** | **<0.001** | **0.011** | **<0.001** | 0.090 | 0.388 |
| MesAC | **0.009** | **<0.001** | **<0.001** | **0.016** | **0.008** | **0.014** | 0.063 |
| CMicAC | 0.092 | **0.049** | **0.040** | 0.091 | **0.043** | **0.012** | 0.059 |
| Silt+clay C | 0.112 | **<0.001** | **0.041** | 0.101 | 0.921 | 0.980 | 0.976 |

C*frac*_1_, Very-labile C fraction; C*frac*_2_, labile C fraction; C*frac_3_*, less-labile C fraction; C*frac_4_*, non-labile C fraction; *TOC*, total organic C, *LI*, lability index, *CMI*, carbon management index; EC, electrical conductivity; *AN*, available-N; *AP*, available-P; *AK*, available-K; *AS*, available-S; *CMacA*, coarse macro-aggregate; *MesA*, meso-aggregate; *CMicA*, coarse micro-aggregate; *WSMacA*, water stable macro-aggregate; *WSMicA*, water stable micro-aggregate; *CMacAC*, coarse macro-aggregated carbon; *MesAC*, meso-aggregated carbon ,*CMicAC* coarse micro-aggregated carbon.
